# Supplementary material for: Rapid trapping and label-free optical characterization of single nanoscale extracellular vesicles and nanoparticles in solution
Source: Light Sci Appl. 2026 Mar 20;15:180. doi: 10.1038/s41377-026-02201-z (PMC13002945; doi:10.1038/s41377-026-02201-z)
Supplement: Supplementary file 1 — SUPPLEMENTAL MATERIAL [file 41377_2026_2201_MOESM1_ESM.docx]

**­ Rapid Trapping and Label-free Optical Characterization of Single Nanoscale Extracellular Vesicles and Nanoparticles in Solution**

Ikjun Hong^1,2^, Chuchuan Hong^3^, Theodore Anyika^1,2^, Guodong Zhu^1,2^, and Maxwell Ugwu^1,4^, James N. Higginbotham^5,6,7^, Jeffrey L. Franklin^5,6,7,8^, Robert Coffey^5,6,7,8^, Justus C. Ndukaife^1,2,6,9*^

*Correspondence: justus.ndukaife@vanderbilt.edu

^1^Vanderbilt Institute of Nanoscale Science and Engineering, Vanderbilt University, Nashville, Tennessee, 37235, United States

^2^Department of Electrical and Computer Engineering, Vanderbilt University, Nashville, Tennessee, 37235, United States

^3^Department of Chemistry, Northwestern University, Evanston, Illinois, 60208, United States

^4^Interdisciplinary Materials Science and Engineering, Vanderbilt University, Nashville, Tennessee, 37235, United States

^5^Department of Medicine, Vanderbilt University Medical Center, Nashville, Tennessee 37232, United States

^6^Center for Extracellular Vesicles Research, Vanderbilt University, Nashville, Tennessee 37235, United States

^7^Epithelial Biology Center, Vanderbilt University Medical Center, Nashville, Tennessee 37235, United States

^8^Department of Cell and Developmental Biology, Vanderbilt University, Nashville, Tennessee 37232, United States

^9^Department of Mechanical Engineering, Vanderbilt University, Nashville, Tennessee 37235, United States

**S1. AC electro osmotic flow simulation**

The AC electro-osmotic (ACEO) flows induced by the micron-scale hole array were simulated in COMSOL Multiphysics. First, the AC electric field distribution within the microfluidic chamber is calculated in a 3D computational domain by solving the Poisson equation given by:

$$\nabla\cdot\mathbf{E}=\frac{\rho}{\varepsilon_{0}}, \mathrm{with} \mathbf{E}=- \nabla V$$

where $\mathbf{E}$ is the electric field, $\rho$ is the local volume charge density, $\varepsilon_{0}$ is the permittivity, and $V$ is the potential. The height of the microfluidic chamber is set to 120 µm, the thickness of the gold film is 15 nm, and the diameter of the micron-scale hole is 15 µm. Using the Electric Current module, the electric potential on the gold film side is set to 0 V, and on the ITO side to 3.53 V, which is corresponds to the root-mean-square value for an applied 10 V peak-to-peak voltage. A periodic boundary condition is applied along the side walls to mimic an infinite array of micron-scale holes, with 18 µm periodicity.

**Figure S1:** a. The in-plane A.C. electric field distribution. b. The out-of-plane A.C. electro-osmotic flow distribution. c. The distribution of the in-plane drag force from A.C electro-osmotic flow. d. The in-plane trapping potential from A.C. electro-osmotic flow e. The out-of-plane particle-surface interaction force along the axial direction.

Following the solution of the electric field distribution within the microfluidic channel shown in Fig. S1a, the Laminar Flow module is then used to calculate the flow velocity distribution for the induced AC electro-osmotic flow. To achieve this, we solved the Navier-Stokes equation in COMSOL Multiphysics given by:

$$\rho_{0}\left[ u\left( \mathbf{r} \right)\cdot\nabla\right]u\left( \mathbf{r} \right)+\nabla\rho\left( \mathbf{r} \right)- \eta\nabla^{2}u\left( \mathbf{r} \right)= \mathbf{F}$$

where $\rho_{0}$ is the density of the fluid, $\rho\left( \mathbf{r} \right)$ is the pressure distribution, $\eta$ is the viscosity of medium, $u\left( \mathbf{r} \right)$ is the fluid velocity, and $\mathbf{F}$ is the body force.

To account for the AC electroosmotic flow, we add a slip velocity boundary condition defined by the Helmholtz-Smoluchowski slip velocity with magnitude that is given by:

$$\boldsymbol{u}_{s}=-\frac{\varepsilon_{w}\zeta}{\eta}\mathbf{E}_{\boldsymbol{\parallel}}$$

where $\mathbf{u}_{s}$ is the velocity of the a.c. electro-osmotic flow, $\varepsilon_{w}$ is the permittivity of the fluid medium, $\zeta$ is the zeta potential, $\eta$is the fluid viscosity, and $\mathbf{E}_{\boldsymbol{\parallel}}$is the tangential component of the electric field established near the surface calculated by the electric current module. The zeta potential was set as -15 mV and the relative permittivity of the fluid was set as 78.

The axial component of the ACEO flow enable fluid circulation and mass conservation in the system as shown in Fig. S1b. The directions of white arrows represent the direction of water flow, and the length shows the magnitude of the ACEO flow velocity. At point A or C, the vertical components of the ACEO flow are pointing downwards, pushing the nanoparticles closer to the gold surface. The in-plane velocity pushes the nanoparticles towards point B. As the in-plane A.C. electro-osmotic flows converge at point B, where the particles are trapped, the particle–surface interacts to enable confinement of the particle in the out-of-plane direction. The particle surface interaction force F_s_ is given by:^1^

$$F_{s}\left( h \right)= \beta\left( h \right)6\pi\varepsilon_{s}\varepsilon_{o}\xi_{p}RE_{pp}\cos\left( wt \right), where \beta\left( h \right)=(h+1.5544R)/(h+0.3R)$$

where $\varepsilon_{s}$ is the permittivity of solution, R is the radius of particle, and $E_{pp}\cos\left( wt \right)$ is the A.C. electric field applied to the electrode with an angular frequency of w, and h denotes the distance from the bottom of the particle to the surface. The particle-surface interaction force (F_s_) is calculated as a function of distance from the surface for a range of particle sizes ranging from 25 nm to 100 nm as shown in Figure S1e. This force balances the drag force from the A.C. electro-osmotic flow in the axial direction to ensure that the particles remain trapped once the particles have been transported to the trapping region, as depicted in Fig. 1b. The balance between the two forces results in stable particle trapping along the vertical axis.

The balance between the two forces results in stable particle trapping along the vertical axis. The in-plane confinement of the particle is mediated by the drag force in the in-plane direction. We have calculated the in-plane drag force and the trapping potential in the in-plane direction as shown in Figure S1c and S1d. For a 100 nm nanoparticle, the in-plane drag force is given by $F=6\pi\eta R\nu$ where $\eta$ is the viscosity of medium, $R$ is the radius of particle, and $\nu$ is the velocity. Here, we used the velocity induced by the in-plane A.C. electro-osmotic flow. Next, the trapping potential energy, as defined by $U\left( x \right)= -\int_{0}^{x} F(l)dl$, is calculated by integrating the provided force along the dotted red line over the hole hole–gold–hole region, as described in Fig. S1c and d. The maximum in-plane trapping potential is approximately -600 k_B_T, which provides a sufficiently deep potential well to stably trap a 100 nm nanoparticle.

**S2. Fabrication procedures**

**Figure S2:** The fabrication procedure outlines the individual steps for the large area fabrication of the nanotweezer device.

**S3. SEM image**

**Figure S3:** The SEM image of the fabricated chip.

**S4. MSD estimation of 100nm, 200 nm, and 300 nm PS particle.**

**Figure S4:** Trapping and label-free detection of 100, 200, and 300 nm PS particles. a. Frame-by-frame images of the trapping experiment for 100, 200, and 300 nm PS particles under a 520 nm laser for ISCAT detection. b, c, d. MSD calculation with linear regression for the estimation of the diffusion coefficient for the three particles.

We trap and characterize the predefined size of particle from 100 nm, 200nm, and 300 nm polystyrene (PS) beads. Figure S4a describes the frame-by-frame events for both trapping and diffusing phases of experiment after the image processing. Trapping mode occurs when the AC field is on, and the particles are under trapped following ACEO flows. Diffusion mode happens when the AC field is off, and the particle undergoes Brownian diffusion. Initially, the 100 nm, 200 nm, and 300 nm mixed particles in the DI water medium freely diffuse during the 0-2.45 s period, while the 520 nm ISCAT laser is prepared for imaging. The concentration of the mixed particles is 2x10^6^ particles$\cdot$mL^-1^, and the field of view (FOV) is 50 µm x 50 µm for the ISCAT detection. When the 3 kHz AC field is turned on, the particles of different sizes become trapped during 2.45-11.67s period, marked by a red, yellow, and green dotted circle in Figure S4a. After trapping, the AC field is turned off to release the particles from the trapping site after 11.67 s.

During diffusion mode, particles of different sizes undergo Brownian dynamics in the medium. Tracking the diffusion of particles in two dimensions with the mean-squared-displacement (MSD) of their trajectories provides the diffusion coefficients for each single particle, following the equation: $MSD\left( \tau\right)= \left\langle\Delta{r\left( \tau\right)}^{2} \right\rangle= \left\langle\left[ r\left( t+\tau\right)-r(t) \right]^{2} \right\rangle$ where $\tau$ is the lag time, $t$ is the designated time, and $r\left( t+\tau\right)$ is the position of particle at $t+\tau$ time frames. Each blue dotted line in Fig. S4b, c, and d represents the calculated MSD plot over the lag time.

The linear fitting slope of the MSD as marked by a red line provides the diffusion coefficient of each particle in the medium. During diffusion mode, particles of different sizes undergo Brownian dynamics in the medium, and the trajectory of each particle is depicted in Fig. 4b of the main manuscript. By tracking the diffusion of particles in two dimensions and calculating the mean-squared displacement (MSD) of their trajectories, we obtain the diffusion coefficients for each particle. The diffusion coefficient is calculated using the equation $D=\frac{MSD\left( \tau\right)}{4\tau}=\frac{\Delta}{4}$ where Δ is the slope of the linear fitting curve. The slopes (Δ) of regression line are 5.4 µm²s^-1^, 7.91 µm²s^-1^, and 16.9 µm²s^-1^, corresponding to estimated PS bead sizes of 151.1 nm, 103.6 nm, and 48.3 nm in radius, respectively.

**S5. Trapping force for Raman analysis**

**Figure S5:** The total force acting on the particle under the operation of Raman trapping with a label free detection.

For the trapping and Raman experiment, a 51 mW, 785 nm laser (IBEAM-SMART-785, TOPTICA) was used with a collimator (F280APC-780, Thorlabs) and focused through a 60×, 1.2 NA water immersion objective lens (Plan APO VC 60×A, Nikon). During Raman acquisition, the 520 nm laser used for interferometric imaging was disabled, while IET trapping remain enabled. To track the Brownian motion, the IET trapping field was turned off and the 520 nm laser was turned on for interferometric imaging.

Once the ACEO flow transports the particle to the stagnation zone, it is stably trapped. Next, the Raman excitation beam is focused on the particle to trap it more securely by the in-plane and out-of-plane optical gradient forces. The Raman spectra were acquired using a spectrometer (Kymera 328i-A, Andor – Oxford Instruments), along with a 785 nm Raman long-pass filter and dichroic mirror set (49950 RT, Chroma). Each spectrum was recorded immediately after the optical trapping event was confirmed under the 520 nm interferometric laser imaging, and a 15-second exposure time was used to ensure sufficient signal intensity.

**S6. Brownian motion of three different EVs.**

**Figure S6:** Tracking the movement of EVs after release from the trapping site to quantify the diffusion coefficient of three different EVs**.**

To independently estimate the EV sizes and generate a calibration plot of their contrast images with respect to size, we released the EVs by temporarily turning OFF the applied AC field and tracking their Brownian dynamics. Figure S6 illustrates the Brownian trajectories of three EVs, with the red, green, and yellow dotted lines tracing their movement in two-dimensional space. Particle localization is tracked over approximately 12 seconds, and the MSD for each EV is shown in Figure 5c, d, and e in the main text.

**S7. Rotational motion of EV**

**Figure S7:** The detection of rotational motion of an EV. The asymmetric shape of the EV induces a non-uniform force on the particle, leading to torque that causes the particle to rotate.

**S8. Label-free trapping and detection of supermeres.**

**Figure S8:** Sequential images showing the trapping and release of supermeres.

**S9. Raman analysis from large EVs.**

**Figure S9:** The label-free detection and Raman analysis from a single large EV. (a, b) The size estimation of large EV1 and EV2 using particle tracking after releasing from the trapping site. (c, d) Raw Raman signal from EV1 and EV2 acquired during trapping and background recorded without trapping.

Raman measurements in Figures 5g and 5h were obtained from the large EVs 1 and 2, as shown in Figure S9. The MSD estimation from the EV Brownian dynamics suggests that the EVs have estimated radius of approximately 394 nm and 185 nm for EV1 and EV 2, respectively. The Raman signals from the single EVs in Figures 5g and 5h were obtained by normalizing the signal against the background, as shown in Figures S9c and S9d. To suppress background signals, we applied first- and second-derivative baseline corrections^2^ using Origin software (OriginLab, USA), which determines a smoothly varying baseline. The baseline B(λ) was obtained by selecting several anchor points over the wavelength range and applying derivative-based fitting to capture the underlying baseline. The first derivative ($I'\left( \lambda\right)$) identifies slowly varying regions, while the second derivative ($I''\left( \lambda\right)$) detects areas of low curvature to ensure that the baseline does not follow sharp peaks. These derivatives guide the fitting to maintain a smooth and accurate baseline. The corrected Raman intensity was then calculated as $I_{corr}\left( \lambda\right)= I\left( \lambda\right)-B(\lambda)$ where $I\left( \lambda\right)$ is the measured Raman spectrum and $B(\lambda)$ represents the estimated baseline derived from derivative guided B-spline fitting, and thereby improves the signal-to-noise ratio (SNR) of the Raman peaks. To further enhance the SNR, confocal Raman microscopy can reduce the collection volume around the trapped particle, thereby improving signal specificity. Although we did not use a confocal setup in this work, implementing a smaller collection volume will be our next step to quantify the single-particle Raman signals with improved signal-to-noise-ratio.

**S10. EVs related Raman spectrum**

| **Frequency (cm^-1^)** | **Biomolecule** | **Assignment** |
| --- | --- | --- |
| 1550–1555 | Proteins | Tryptophan (W3) |
| 1515–1540 | Carotenoids | Polyene ν(C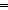C) |
| 1450–1490 | Nucleic acids | Purine adenine, guanine ring |
| 1435–1465 | Proteins | Backbone δ (CH_2_, CH_3_) |
|  | Lipids | δ (CH_2_, CH_3_) in acyl chain |
| 1300–1350 | Proteins | Backbone δ (C_α_H), ν(C_α_–C) |
| 1295–1305 | Lipids | δ (CH_2_) in acyl chain |
| 1260–1270 | Lipids | δ (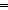CH_2_) in acyl chain |
| 1230–1305 | Proteins | Amide III: ν (C–N) + δ (NH) |
| 1207–1210 | Proteins | Phenylalanine (F3), Tyrosine (Y3) |
| 1175–1177 | Proteins | Tyrosine (Y4) |
| 1155–1160 | Carotenoids | Polyene ν(C–C) |
| 1050–1160 | Proteins | Backbone ν(C_α_–N, C_α_–C, C–N) |
| 1300–1350 | Lipids | ν(C–C) in acyl chain |
| 1032 | Proteins | Phenylalanine (F4) |
| 1012 | Proteins | Tryptophan (W6) |
| 1004 | Proteins | Phenylalanine (F5) |
| 930–960 | Proteins | α-Helix backbone ν(C–C_α_–N) |
| 878–880 | Proteins | Tryptophan (W7) |
| 820–900  810–836 | Phospholipids  Nucleic acids | ν(O–C–C–N^+^), ν(C_4_–N^+^)  Phosphodiester ν_s_(O–P–O) |
| 758–759  725–751  717  700–704 | Proteins  Nucleic acids  Phospholipids  Lipids | Tryptophan (W8)  Adenine  ν_s_(C–N^+^)  Cholesterol |
|  |  |  |

ν = Stretching mode, δ = deformation mode; This EV-related Raman table is quoted from Kruglik, Sergei G., et al. 'Raman tweezers microspectroscopy of circa 100 nm extracellular vesicles.^3^

**S11. EV and supermere preparation.**

EVs and supermeres were isolated from a hollow fiber bioreactor (FiberCell, New Market, MD) conditioned media from DiFi cells as described in ^4^, except that the second higher speed spin was at 2,500 x g not 1363 x g. Supermere and small EV pellets where purified as described in^5^.

**S12. The local heating simulation.**

**Figure S10: a.** The temperature distribution under 520 nm incidence on a glass substrate. b. The temperature distribution under 785 nm incidence on an Al_2_O_3_ substrate.

We used the local heat source defined by^6^

$$Q\left( r \right)=P_{o}A\frac{\alpha_{Au}}{2\pi\sigma^{2}}e^{-\frac{x^{2}+y^{2}}{{2\sigma}^{2}}}e^{-\alpha_{Au}z}$$

contrast term can be described as

$C=\left( \frac{\left| s \right|}{t} \right)^{2}$+$2\frac{\left| s \right|}{t}=\left( \frac{1}{t} \right)^{2}\sigma_{scat}+2\left( \frac{1}{t} \right)\sqrt{\sigma_{scat}}$

, where the $\sigma_{scat}$ is the scattering cross-section from the particle.

$P_{o}$ is the laser power, $A$ is absorptance, $\alpha_{Au}$is the attenuation coefficient, and the Gaussian beam’s waist radius is defined as $w=2\sigma$. We set $P_{o}$ to 0.5 mW, the absorptance to 0.23, and the attenuation coefficient to 5$\times$10^-7^m^-1^, and $\sigma$ to 50 um, and the thermal conductivity of glass to 1.5 Wm^-1^K^-1^. The calculated temperature increase is ~0.09 K, which is negligible given the low laser intensity used in the experiments.

While the interferometric scattering measurements can be readily performed with the fabricated chip on a glass substrate, for the Raman measurements, we switched to a sapphire (Al_2_O_3_) substrate, which has a higher thermal conductivity of 40 Wm^-1^K^-1^ compared to that of glass (1.5 Wm^-1^K^-1^), to mitigate the local heating effect with the 51 mW 785 nm Raman laser. The temperature distribution under the 785 nm laser illumination was simulated using a beam with a 51 mW power. We set $P_{o}$ to 51 mW, the absorptance to 0.05, and the attenuation coefficient to 7.6$\times$10^-7^ m^-1^, and $\sigma$ to 0.56 um, and the thermal conductivity of sapphire to 40 Wm^-1^K^-1^. The temperature rise above the 200 nm surface, where the trapping is expected, is ~10K, which represents a non-negligible increase for Brownian dynamics analysis. The Brownian motion trajectories presented in the Supplementary Information S9 were recorded before conducting the Raman experiments, as the environmental temperature is a critical parameter for accurate MSD calculations.

**Movies:**

Supplementary video 1: Rapid and parallel trapping of a 300 nm PS bead for the raw video.

Supplementary video 2: Rapid and parallel trapping of a 300 nm PS bead after the background subtraction.

Supplementary video 3: 100nm, 200 nm, and 300 nm PS beads trapping and releasing after the background subtraction.

Supplementary video 4: EVs trapping and release after the background subtraction.

Supplementary video 5: EVs rotational motion after the background subtraction showing that our approach can detect irregular shaped particles that are non-spherical such as two EVs fused together.

Supplementary video 6: Supermeres trapping and releasing after the background subtraction.

**Reference**

1. Gong, J. & Wu, N. Electric-field assisted assembly of colloidal particles into ordered nonclose-packed arrays. *Langmuir* **33**, 5769–5776 (2017).

2. Lieber, C. A. & Mahadevan-Jansen, A. Automated method for subtraction of fluorescence from biological Raman spectra. *Appl Spectrosc* **57**, 1363–1367 (2003).

3. Kruglik, S. G. *et al.* Raman tweezers microspectroscopy of circa 100 nm extracellular vesicles. *Nanoscale* **11**, 1661–1679 (2019).

4. Hong, I. *et al.* Anapole-Assisted Low-Power Optical Trapping of Nanoscale Extracellular Vesicles and Particles. *Nano Lett* (2023).

5. Zhang, Q., Jeppesen, D. K., Higginbotham, J. N., Franklin, J. L. & Coffey, R. J. Comprehensive isolation of extracellular vesicles and nanoparticles. *Nat Protoc* **18**, 1462–1487 (2023).

6. Yang, S. & Ndukaife, J. C. Optofluidic transport and assembly of nanoparticles using an all-dielectric quasi-BIC metasurface. *Light Sci Appl* **12**, 188 (2023).
